# CUDT: A CUDA Based Decision Tree Algorithm

**DOI:** 10.1155/2014/745640

**Published:** 2014-07-22

**Authors:** Win-Tsung Lo, Yue-Shan Chang, Ruey-Kai Sheu, Chun-Chieh Chiu, Shyan-Ming Yuan

**Affiliations:** ^1^Department of Computer Science, Tung Hai University, Taichung 40704, Taiwan; ^2^Department of Computer Science and Information Engineering, National Taipei University, New Taipei 23741, Taiwan; ^3^Department of Computer Science, National Chiao Tung University, Hsinchu 30010, Taiwan

## Abstract

Decision tree is one of the famous classification methods in data mining. Many researches have been proposed, which were focusing on improving the performance of decision tree. However, those algorithms are developed and run on traditional distributed systems. Obviously the latency could not be improved while processing huge data generated by ubiquitous sensing node in the era without new technology help. In order to improve data processing latency in huge data mining, in this paper, we design and implement a new parallelized decision tree algorithm on a CUDA (compute unified device architecture), which is a GPGPU solution provided by NVIDIA. In the proposed system, CPU is responsible for flow control while the GPU is responsible for computation. We have conducted many experiments to evaluate system performance of CUDT and made a comparison with traditional CPU version. The results show that CUDT is 5∼55 times faster than Weka-j48 and is 18 times speedup than SPRINT for large data set.

## 1. Introduction

With the advances of Internet-Of-Thing and sensing technology, there are increasingly sensing devices which comprise sensors and actuators, and data processors have been deployed to sensing, capturing, and collecting real world environmental data. The European Commission [1] has predicted that the present “Internet of PCs” will move towards “Internet of Things” in which 50 to 100 billion devices will be connected to the Internet by 2020 and it is expected that the generated data will reach 35 ZB in 2020 [2]. To process and employ the tremendous data, a well-designed high-performance computing environment with excellent data mining technology can accelerate the data processing latency.

In addition, the GPU (graphics processing unit) is a specialized electronic circuit designed to rapidly manipulate and alter memory to accelerate the creation of images in a frame buffer intended for output to a display. In recent years, the general-purpose computing on GPU (GPGPU for short) has become popular due to its highly parallelization and powerful computing ability of float point. Some documents show that the computing power of GPUs can now vastly exceed traditional CPU [3–5]. More and more nongraphic applications which needed amounts of computation are employed on GPU.

Data mining on various environments and data sources is an important technique in recent years [6–9]. Decision tree learning is a famous learning method commonly used to data classification in data mining [6, 7, 10–12]. It is one of the most successful techniques for supervised classification learning. Many data mining software packages provide implementations of one or more decision tree algorithms. Recently, many researches were focusing on improving performance of decision tree [13–15]. However, those algorithms are developed and run on traditional distributed systems. In the [16], authors presented two basic parallel formulations of classification decision tree learning algorithm based on induction. In the work, experimental results on an IBM SP-2 demonstrate excellent speedups and scalability. Obviously the latency could not be improved while processing huge data generated by ubiquitous sensing nodes without new technology help [17].

In order to improve data processing latency in huge data mining, in this paper we design and implement a new parallelized decision tree algorithm on a CUDA (compute unified device architecture), which is a GPGPU solution provided by NVIDIA [18–20]. By leveraging the existing CUDA components, such as prefix-sum and parallel sorting, our proposed CUDT (CUDA based decision tree algorithm) system performs well and gets major performance improvement than sequential decision tree algorithms. We have conducted many experiments to evaluate system performance of CUDT and made a comparison with traditional CPU version. Comparing to the famous Java open source project Weka, the CUDT has 5~55 times faster than Weka in similar data accuracy level. Comparing to the best optimized SPRINT [14, 21], CUDT has maximum 18 times faster than SPRINT. The experiment result shows that CUDT gets remarkable performance improvement than other decision tree implementations.

The rest of the paper is organized as follows.  Section 2 is background and related works. We will present the CUDA architecture and some important parallel primitives. The related works show the recent researches of decision tree on GPUs.  Section 3 is our system architecture in detail.  Section 4 is the evaluation of our algorithm. The last part of the paper is the conclusion and future work.

## 2. Background and Related Work

### 2.1. Decision Tree

A decision tree is a decision support tool that uses a tree-like graph or model of decisions and their possible consequences, including chance event outcomes, resource costs, and utility [6, 7]. It is commonly used in machine learning or data mining and shows the one-way path for specific decision algorithms. A decision tree consisted of two kinds of nodes. An internal node represents a decision rule, and a leaf node shows the result of a decision.

**Figure 1:**
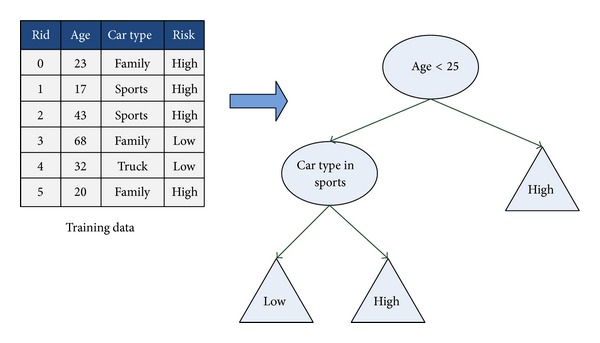
An example of decision tree.

Figure 1 shows an example of a decision tree used for classifying training data into groups of high or low risk. For the root node, the decision rule is whether the age of the input training data that is smaller than 25 or not. For the first row of the training data, the age is 23 and is smaller than 25. The result of the risk level would be high. Similarly, for the row of the 3rd one, the age is 43, and it is larger than 25. The decision path will go to the next internal node to check the car type. Again, the car type is sports, and it matches the decision rule. The result of the decision path would be directed into the leaf node of high risk level.

### 2.2. Prefix-Sum

Prefix-sum is a very important building block of parallel algorithms, and it is implemented by a* scan* function in CUDA environments. Many applications such as sorting, lexically comparing strings, and evaluated polynomial can be implemented by the* scan* function [22, 23]. A definition of the prefix-sum is shown in Algorithm 1. The prefix-sum element will be the result of interoperations between all previous elements.

Prefix-sum makes no sense in sequential algorithms but it is very important in parallel algorithms. Both CUDA SDK and CUDPP (CUDA Data Parallel Primitives Library) have a* scan* function for it. The CUDPP sorting algorithm is a very high-performance function for CUDA radix sort. The* scan* function is the major backbone of CUDPP sorting function, and each round of sorting is building on the prefix-sum function [22]. In our proposed decision tree algorithm, the parallel prefix-sum function of CUDPP is also heavily used in many system components.

### 2.3. SPRINT: A Scalable Parallel Classifier for Data Mining

**Figure 2:**
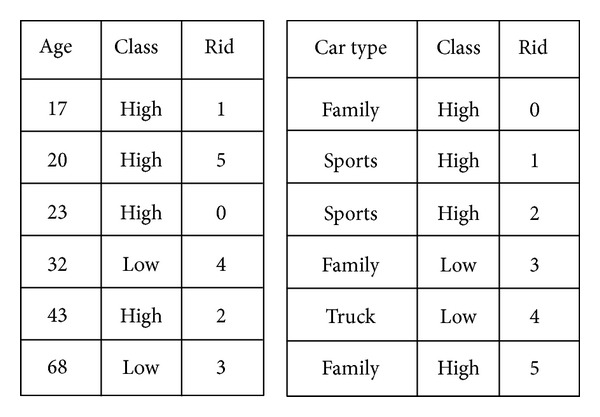
Sample of SPRINT attributes list.

**Figure 3:**
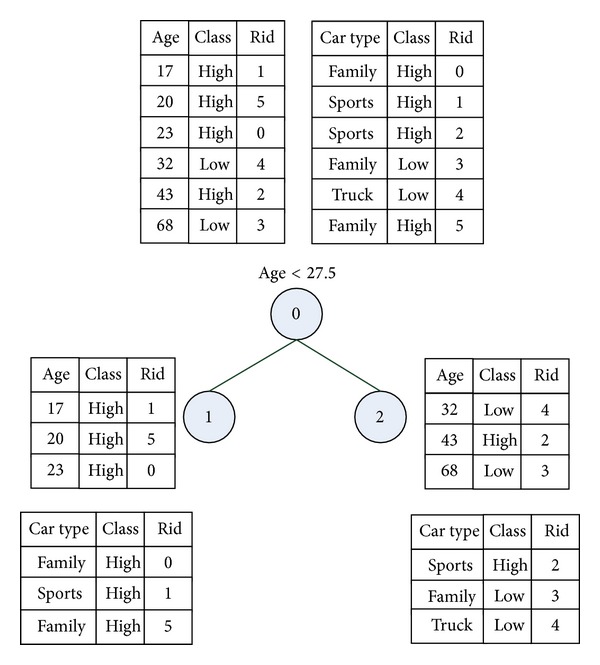
Splitting an attributes list into disjoint subspaces in SPRINT.

SPRINT is a classical algorithm for building parallel decision trees, and it aims at reducing the time of building a decision tree and eliminating the barrier of memory consumptions [14, 21]. Traditionally, decision tree algorithms need several passes to sort a sequence of continuous data set and will cost much in execution time. In contrast to traditional algorithms, the SPRINT just needs one pass to sort a sequence of data by leveraging its own data structure, called attributed list.  Figure 2 shows an example of attribute list. The left one is an attribute list of continuous attributes, and the right one is an example of categorical attributes. An attribute list is composed of three arrays. The first array is the attribute value, the second one is the class label of the record, and the last one is the index of records. It is obvious that each attribute list is independent, so that we can sort each continuous attribute in one pass, and does not need extra sorting phases. The key of a high-performance decision tree is how to find a data point to split attributes into subsets. SPRINT has a good strategy for splitting attributes into disjoint subsets. In its split stage, each list will be split into two disjoint subspaces.  Figure 3 shows an example of splitting. This mechanism reduces the overhead of sorting but increases the overhead in splitting the attribute lists. However, the new overhead is smaller compare to repeat sorting.

**Figure 4:**
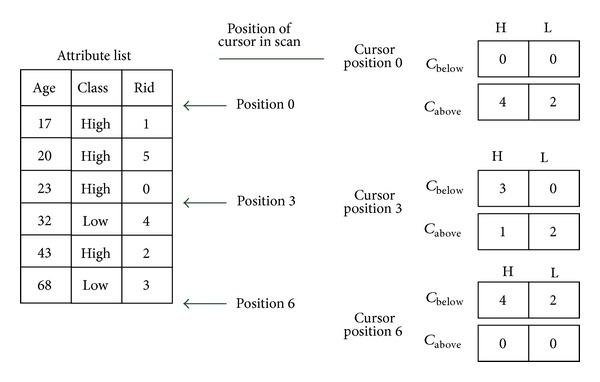
*C*_above_ and *C*_below_ of SPRINT [14].

In order to find the best split point, the SPRINT algorithm needs to calculate the criteria of splitting. SPRINT has two different approaches for each attribute. For continuous attribute, SPRINT uses two histograms, denoted by *C*_below_ and *C*_above_, to capture the class distribution of the attribute records at a given node.  Figure 4 shows an example of the two histograms. *C*_below_ records the sum of each class number before current data and *C*_above_ records the sum of each class number after current data.

**Figure 5:**
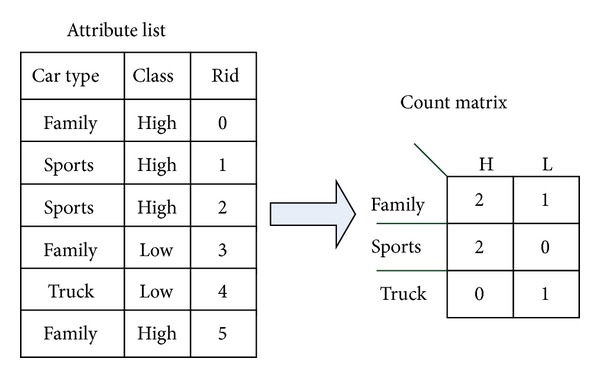
Count matrix of SPRINT [14].

For categorical attribute, SPRINT uses a histogram called “count matrix” to split attributes.  Figure 5 shows an example of count matrix. Each entry of count matrix records a distributed class value of the attribute. After finishing the calculation of class distribution, we have all information of calculating split criteria.

In parallel version SPRINT, it partitions the attribute lists into several subspaces of the same size. Each processor calculates the local class distribution and exchanges with each other to get the global class distribution. After getting the global class distribution, each processor calculates the local split criteria of all possible split points. For continuous attributes, the possible split points of an attribute are all different value points. For categorical attributes, the number of possible split points is equal to the number of different values of the attributes. After finishing the local split criteria, each processor finds the local best point. In order to get the global best split point, all processors communicate with each other to find the best spilt points.

## 3. System Design

By leveraging the idea and advantages of the parallel SPRINT algorithm, the proposed CUDT algorithm and the prototype of the implementation are shown in the following sessions. Firstly, the system overview and the flowchart of the prototype are illustrated, and then the details of how to find a splitting data point and the algorithms for splitting attribute list are described in the next session.

### 3.1. System Overview

**Figure 6:**
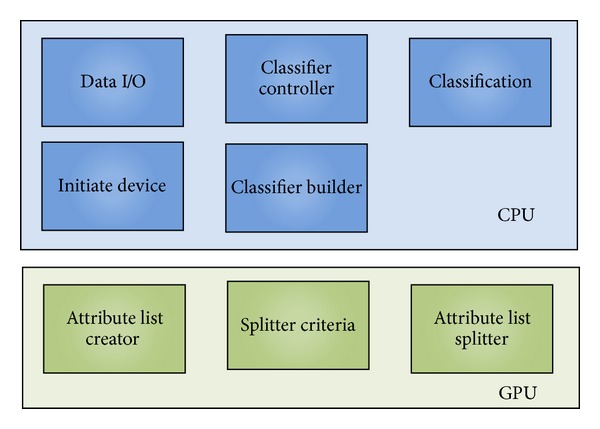
The CUDT system components.

The principle of CUDT is dispatching flow control, I/O handling, and communication tasks to CPU and on the other hand assigning computing intensive jobs to GPU.  Figure 6 shows the components of CUDT system. The blue parts are running on CPU, and they are data I/O, classifier controller, classification, initiate device, and classifier builder components. As for the green parts running on a GPU, there are three components, and they are create attribute list, split criteria, and split attribute lists.

In contrast to common CudaRF functions which parallelize both the training and the classification phase, the CUDT focuses only on how to process the computation of splitting nodes in parallel. Although it gains nothing from the parallelism of building multiple trees, the CUDT increases much improvement of system scalability and performance while the data set is huge.

### 3.2. CUDT Flowchart

**Figure 7:**
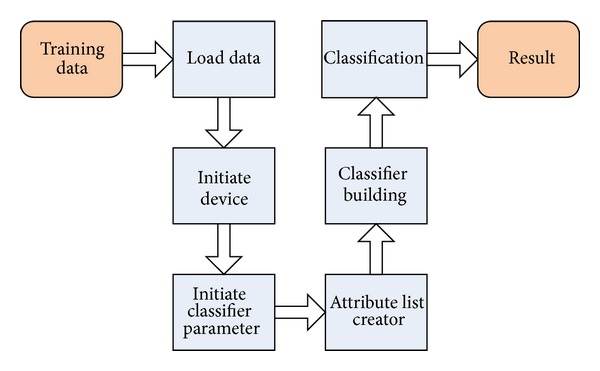
Flowchart of the proposed CUDT algorithm.

As shown in Figure 7, there are seven major steps in the CUDT system.

**Figure 8:**
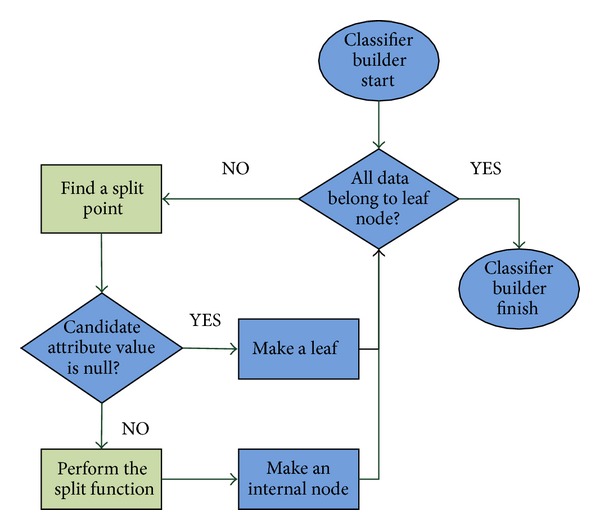
Flowchart of the classifier building phase.

1. Training and testing data are loaded to host memory from disks.
2. Initialization of the device includes query device information, allocation memory space, and copy of training data into device.
3. In this step, the system will set up some parameters from user. For instance, the minimum numbers of data of a leaf are, the maximum depth of the classifier is.
4. Creating attribute lists in device. We will move each attribute to corresponding position. After finishing the data movement, we would sort all attribute lists in devices.
5. Step 5 is the most important one of the system. Instead of using the recursive model of decision tree building algorithm, we use an iterative breadth first scheme for our proposed system. Host plays a role of a manager and is in charge of working flow of the whole system.  Figure 8 shows a flowchart of building classifier.
6. The classification is performed on the host. In other words, the process of classification is executed sequentially.
7. The results are presented on hosts.

More details of the flowchart of building classifier are identified as follows. The system will execute loop until all data have belonged to leaf. For a segment of data, the system would check if all data of this segment have the same class label, which is positive or negative in our system. It makes a leaf node if all classes of data are the same or process the finding of a split point process of the segment. A leaf node denotes a result of classification. The data would be classified as the class of leaf nodes if it stops at this node in classifying process. After finding a candidate split point, we need to split the attribute list and make an internal node. An internal node could be thought of as a rule which decides the path to classify the data.

In the next section, we will describe in more detail all system components, for instance, how the attribute list is created in step 4 and how to find a candidate split point and split attribute lists in step 5. It also shows how CUDT applies those parallel primitives and how it employs the computation power of GPU to our system.

### 3.3. System Components

#### 3.3.1. Load Data and Initiate Device

The CUDT flowchart starts with the host reading input data from a disk. After loading data from the disk, the system will allocate the space of the device memory to store the data. The allocation includes entire training data, the space of attribute lists, and some internal buffer inside of the device.

#### 3.3.2. Initiate Classifier Parameters

After finishing the allocation of device memory, we set the user-defined parameters of CUDT, for example, the minimum size of data of a leaf, the maximum depth of the decision tree, the type of classification evaluation, and the like.

#### 3.3.3. Create Attribute Lists

There are two parts of creating attribute lists. The first one is moving the data to its corresponding list. After finishing the data movement, we need to sort all attribute lists. There is a well-known CUDA library which is called CUDPP. CUDPP offers a serial efficient library to developers. Several important algorithms are implemented in those libraries, for instance, parallel prefix-sum and parallel sorting. However the CUDPP has a wonderful parallel radix sort algorithm; the sorting algorithm is not suitable for our system. The sorting algorithm of CUDPP can sort two 1D arrays as input, the first is called key array, and the second one is called value array. The key array would be sorted and the position of each element of value array would be changed according to its corresponding key element. It is called a key value pair sorting. The sorting algorithm of CUDPP only supports a key value pair sorting, but we have two values to one key consisting of the* attribute value* field and* rid *and* class label*. Based on the above arguments, we modified the CUDPP sorting algorithm into one key of two values. In order to get the best performance, we modified the sorting algorithm from the CTA level to public interface level [24].

#### 3.3.4. Classifier Builder

There are two important functions provided by the building classifier. The first one is “finding split point” which performs tasks of finding the candidate split point and attribute. The second one is “split attribute lists” which would be executed after finding a valid spit point.

*Split point finder* and the* attribute list splitter* components are the most important functions in the CUDT system, and there will be algorithms of more details shown in the next section.

#### 3.3.5. Classification

The tree is stored in host memory. The reason of constructing the classifier in host is the consideration of scalability. If the size of a tree is greater than the memory size of device, there are no ideals to maintain the tree in device memory. Our algorithm is designed for general cases. The system should be easy to scale in bigger data set. That is why we choose the policy. Since the tree is stored in host memory, the classification is processed in host side. All data are tested in sequence.

### 3.4. Algorithms of Finding Split Points

While scaling up a decision tree, the goal at each node is to find the best attribute and split point to divide the training data into several subsets. The value of a split point depends on how well it separates the class distributions. There are many split criteria that have been proposed in the past. For better system performance considerations, we adopt the Gini index [25] as the splitting criteria for the CUDT system. Firstly, let us consider how it works for sequential algorithms. For the sequential version, the process needs to scan an attribute list to a class distribution table. After finishing filling the table, the process has all information to calculate the Gini index and find the best split point of this attribute. However, it only processes one attribute at a time. We need to calculate all attribute lists and find the best among them at one pass.

**Figure 9:**
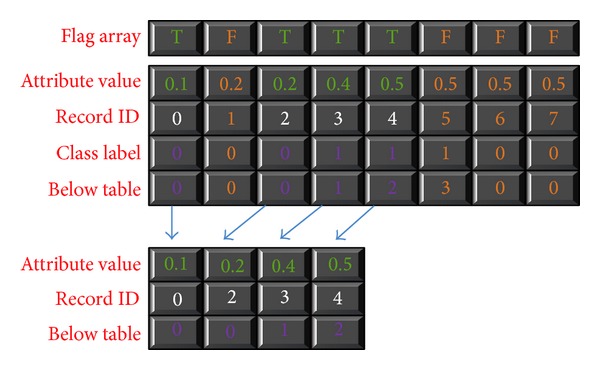
Example of compact function.

In the CUDT system, firstly, the system needs to record the class distribution into below table and save the number of total positive classes. For continuous attribute, the candidate split points are midpoints between every two consecutive attribute values. It is obvious that there are many redundant elements of the below table, so the system needs to remove unnecessary data from the histogram. The procedure is called* compact*.  Algorithm 2 shows the algorithm of compact. The compact needs a flag array and other arrays as input. The value of flag element is “0” or “1.” “0” means that the correlative payloads are true elements. True elements should be reserved in final output. The algorithm first scans the flag array to get the positions of true elements. After getting the position, the threads with true elements would put the elements into their positions. Each thread loops several times to put all payloads into correct address.  Figure 9 is an example of compact. After getting valid split points of all attributes, the system will calculate the splitting criteria of all possible split points. Since the class distribution table has all class information of the data segment, we can calculate the Gini index of all possible split points.

**Figure 10:**
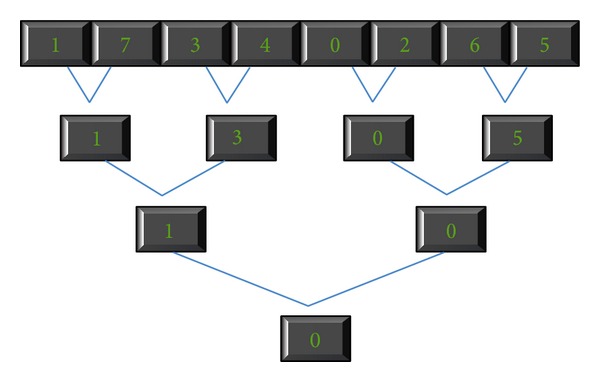
Example of Reduce Function.

The final step of this algorithm is to find the best point from the possible splitting points. There is a parallel primitive called “*reduction*.” A brief description of* reduction* is that many parallel threads generate a single result.  Figure 10 shows how to reduce an array to find a minimum value. We use the CUDPP prefix-sum library of CTA level to implement the* reduction*. The algorithm of* reduction* is described in detail in Algorithm 3. After finding the best split point whose algorithm is shown in Algorithm 4, devices will upload the information to hosts. After getting the data, hosts can set up the information of children of this node and split the attribute lists.

### 3.5. Algorithms of Splitting Attribute List

In traditional algorithms of building decision tree, the split attribute lists do not need extra work since all data are stored in order. They label the split points of the data segment of this node. However, this would not work in the CUDT system since we partition an attribute as a single list. The different lists may have different data in the same position. Hence, we need an extra operation to split attribute lists. Although the system needs some extra executing time in splitting attribute lists, CUDA architecture is suitable for binary split operations. It reduces the overhead caused by data splitting.

**Figure 11:**
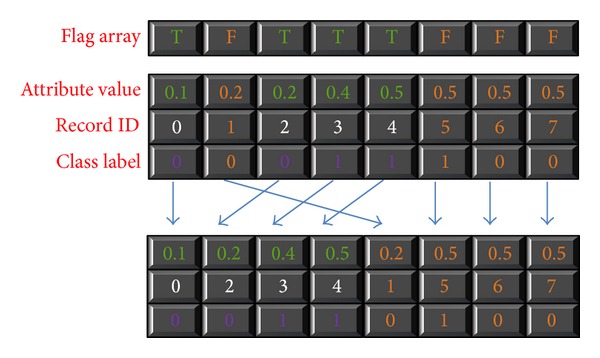
Example of split attribute lists.

Algorithm 5 shows the algorithm of* splitting the attribute lists* function. Partitioning the attribute list of the winning attribute is trivial. It just sets the split index of winning split point of this attribute to node. Handling the winning attribute is easy; however, we still need a mapping table for rid and subtrees. The CUDT system uses a table to store these mapping relationships. A record is assigned to the left partition if its value is smaller than the split point or it will be assigned to the right partition. After finishing splitting the winning attributes, the algorithm will keep work in the other attributes by picking another element from the mapping table. Similar to the process of finding a split point, the system splits all attributes in one pass. A thread handles a record of an attribute list, finds the location of the record, and stores the result into a side array. We recall a CUDPP parallel prefix-sum to calculate the side array, then moving all data of the segment into a buffer and performing a partition function.  Figure 11 shows an example of splitting attribute lists.

The basic ideal of partition is partitioning an array into two disjoint subspaces.  Algorithm 6 shows* partition* algorithm in detail. The algorithm will partition the input data into two subspaces according to the flag array. The element will be assigned to left group if its flag is 0 or it would be assigned to right group. The algorithm first complements flag array and prefix-sum to get a false array. The total number of the false elements is recorded. The next step of the algorithm is calculating the index of each element after partitioning. The index of an element is calculated if it is a false element. If it is a true element, the index will equal “the original index − above index + total number of false elements.” The final step is moving the elements to this position of the partition.

## 4. System Evaluation

Our goal of the design of CUDT system is to propose a high-performance decision tree algorithm. Although the results show that the CUDT is also an accuracy-acceptable system, we focus our experimental evaluation on performance issues. For comparison purpose, we leverage the open source Weka (http://www.cs.waikato.ac.nz/ml/weka/) to be one of the target platforms for performance evaluations. Another benchmark we compared it with is the sequential SPRINT. Because the CUDT is also a type of SPRINT running on GPU, we also evaluate all components for CUDT and SPRINT.

### 4.1. Evaluation Environments

We adopt Intel Core 2 Quad Q6600 and Geforce 9800GT as our computation platform. The configuration information is described as follows. Our host is Intel Core 2 Quad Q6600 with 4 cores. Each core has a clock rate with 2.4 GHz. Our device is GeForce 9800GT which has 14 multiprocessors which are called MPs. A MP has 8 CUDA cores. There are 112 CUDA cores in total. The CUDA version is the version of the device driver. There are many new features in the newer version. The newest version of CUDA is 4.0RC. However, the features of CUDA 4.0 only impact the recent generation of the GPU. There is no influence of our device. The compute capability means difference generation of CUDA GPUs [18, 19]. Although our device is not as good as CPU, our system shows a good speedup on 9800GT.

Table 1 shows the details of test data set. There are three data sets in our evaluation environment. The spambase is a collection of mail data which has 58 continuous attributes of features of spam mails. A categorical attribute denotes spam and nonspam. The number of data is 4601. The second data set is Magic Gamma Telescope. It is physical data of high energy gamma particles. There are 19020 data of this data set. It has 11 continuous attributes of each record. A categorical attribute denotes the data into two classes. The final data is also physical data. It has 51 attributes and 130065 data numbers.

### 4.2. Evaluation Analysis

Tables 2, 3, and 4 show the result of three algorithms. The tables include total cost time of building classifier, the accuracy of the classifier, and the size of classifier. We use cross-validation to evaluate the accuracy of our system. It means that we use all training data as the test data. It shows that the accuracy of our system is very close to Weka-j48 and the execution time is shorter than both Weka and SPRINT algorithms. The tree sizes are the same of SPRINT and CUDT since we use the same criteria for data splitting.  Figure 14 is the speedup of the classifier builder step in CUDT system.

**Figure 12:**
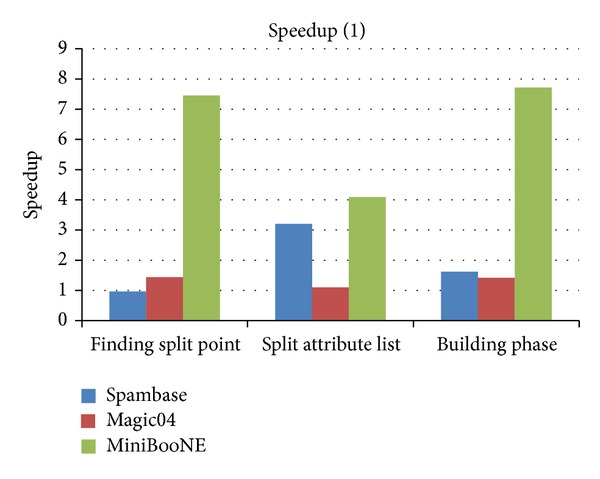
Speedup of each component (1).

**Figure 13:**
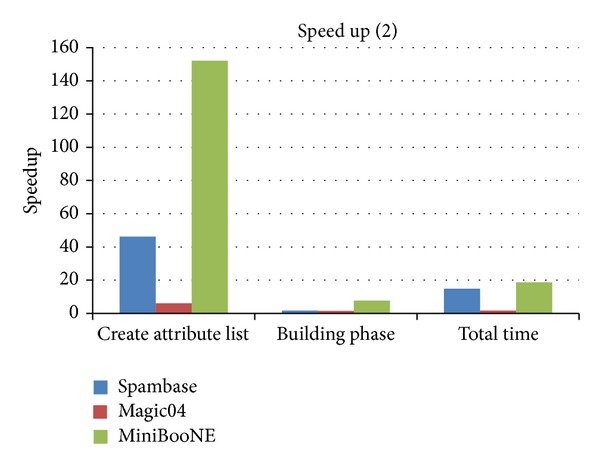
Speedup of each component (2).

**Figure 14:**
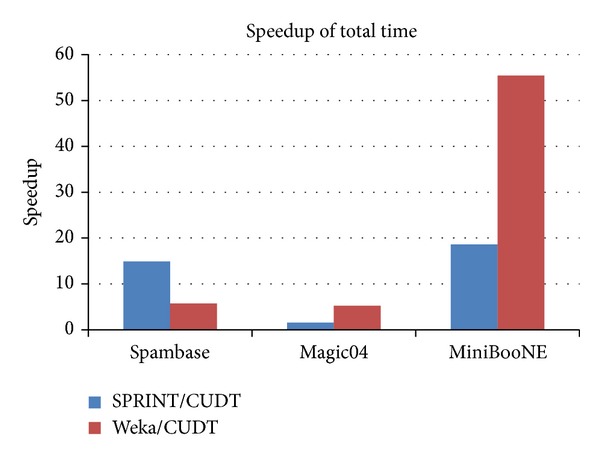
Speedup of classifier builder.

In order to evaluate the speedup of all components of CUDT, we compare it with SPRINT in detail in Table 5 which shows the execution time of each component of CUDT and SPRINT.  Figure 12 shows the speedup of each component. The time of building a tree is the sum of finding split point and splitting the attribute lists. Since the speedup of creating attribute lists is much higher than other components, we show it separately on Figure 13. Total time is the sum of building phase and creating attribute lists. We can see that the CUDT system performs very well for large data set.

The performance of creating attribute lists is also very good. It can achieve 14x times faster than SPRINT. However, the other components of CUDT are not as good as creating attribute lists. Because both the execution and communication time are correlated to the size of tree size, the performance downgrades slightly when the tree size increased. The CUDT system generates too many nodes that increase the execution time when building a decision tree. The second cause of CUDT performance downgrading is the size of data. The larger the size of data, the more the time consumption for data value calculation. The last reason is that the increasing of nodes per level will cause the data swap between CPU and GPU which takes much instruction execution time. We will also discuss those issues in the following sections.

### 4.3. Evaluation for Difference Levels

**Figure 15:**
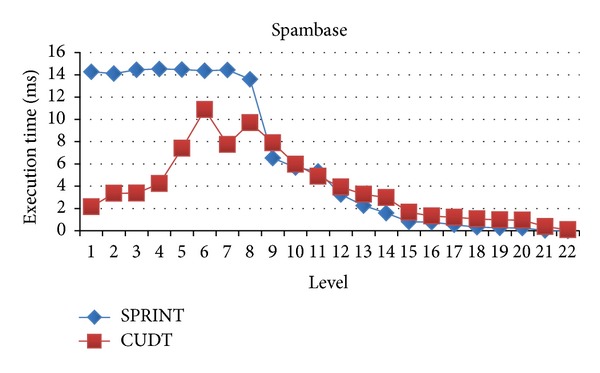
Execution time of each level on spambase.

**Figure 16:**
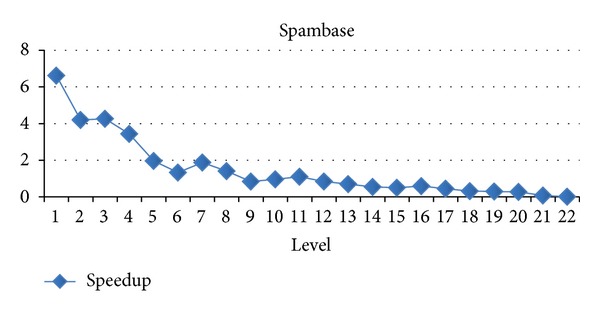
Speedup of level of spambase.

**Figure 17:**
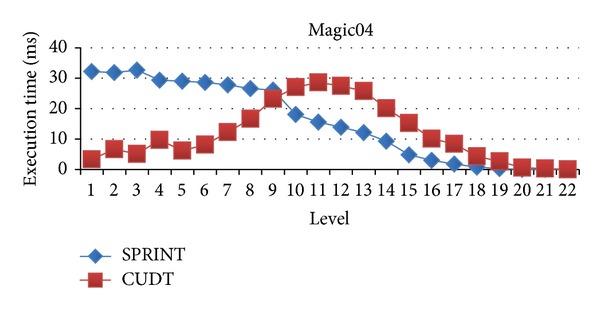
Execution time of each level on Magic04.

**Figure 18:**
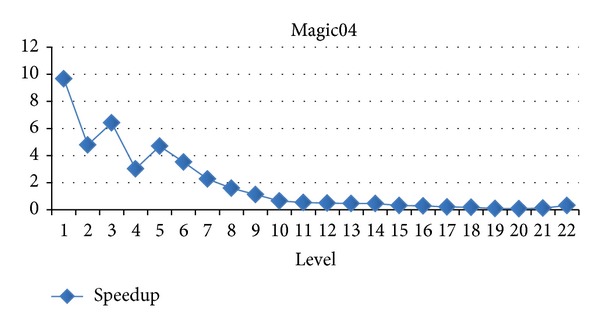
Speedup of level of Magic04.

**Figure 19:**
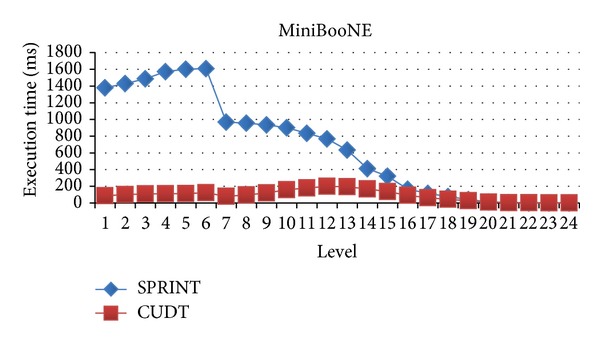
Execution time of each Level on MiniBooNE.

**Figure 20:**
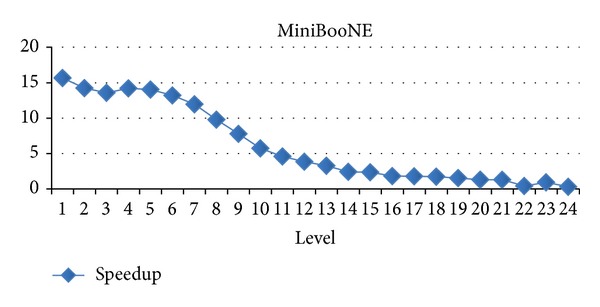
Speedup of level of MiniBooNE.

In this section, we will evaluate each level of decision tree building. Since create attribute lists component performs very well, we focus the discussion on the tree building phase. Figures 15, 16, and 17 show the execution time of each level of CUDT and SPRINT. Figures 18, 19, and 20 show the speedup of each level. The best speedup is always on the first level. There are two reasons for the observations. The first one reason is that the active data size is always large on the first level. A GPU device is composed of many weak cores. Large data can exert the computation power of GPU. This is why CUDT always performs better than SPRINT on the first level. The second one reason is that the increased node numbers on each level also cost additional communication efforts between CPU and GPU. Since we only parallelize the computation of creating a single node, the building phase makes a tree iteratively. Besides, we need to upload some data from GPU to CPU after finding the split points. The increasing of tree nodes will also increase the data movements between CPU and GPU. Due to the same reason, the following level's performance is not as good as the first level.

**Figure 21:**
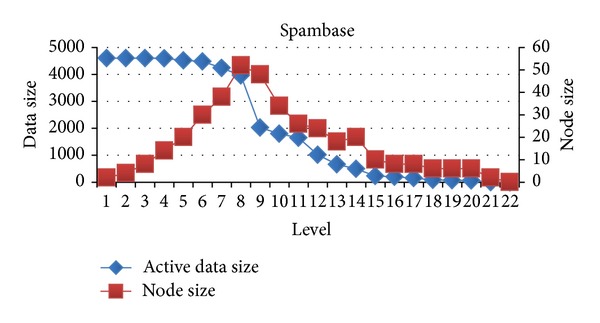
Active data size versus node size on spambase.

**Figure 22:**
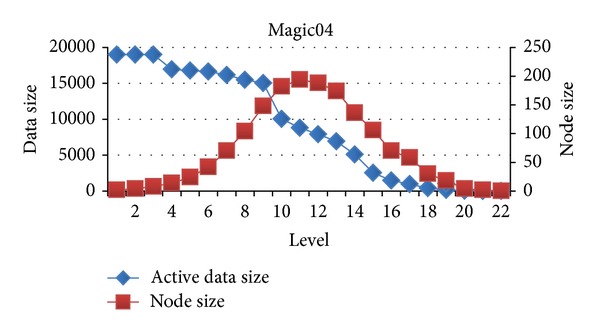
Active data size versus node size on Magic04.

**Figure 23:**
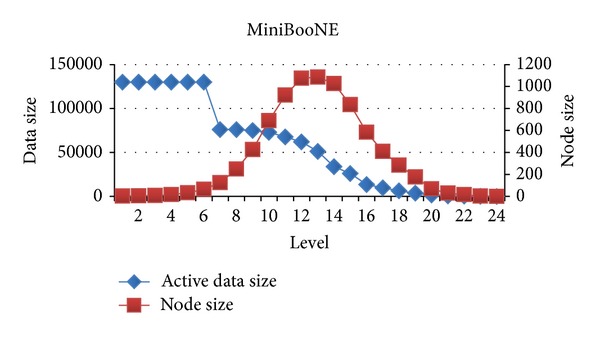
Active data size versus node size on MiniBooNE.

Similar to the above reasons, the upper levels have better speedup than the lower levels. There is a performance turning point between CPU and GPU. Figures 21, 22, and 23 show the relationship between node size and active data size. The blue line presents active data size. The red one is the size of nodes on the level. We can see that the trend of execution time of CUDT is very close to the size of node on each level. It shows that the CUDT system is more sensitive with node sizes prior to the data size.

## 5. Conclusions and Future Works

Using GPU for solving problems with high density computation normally brings remarkable improving of performance. Of course, the precondition is that these problems should be able to be solved in parallel. Many machine learning algorithms have been developed on CUDA GPUs. They also show performance improvement comparing to the implementation of CPU. In this paper, we studied the background of existing decision tree algorithm and CUDA programming model. Based on the knowledge, we proposed a new parallel decision tree algorithm base on CUDA. By leveraging the existing CUDA components, such as prefix-sum and parallel sorting, our proposed CUDT system performs well and gets major performance improvement than sequential decision tree algorithms. Comparing to the famous Java open source project Weka, the CUDT has 5~55 times faster than Weka in similar data accuracy level. Comparing to the best optimized SPRINT, CUDT has maximum 18 times faster than SPRINT. The experiment result shows that CUDT gets remarkable performance improvement than other decision tree implementations.

The major problem of CUDT algorithm is that redundant nodes not only hurt performance of building phase but also reduce the accuracy of results. The further studies of this system have to focus on the issue of tree size.

## Figures and Tables

**Algorithm 1:**
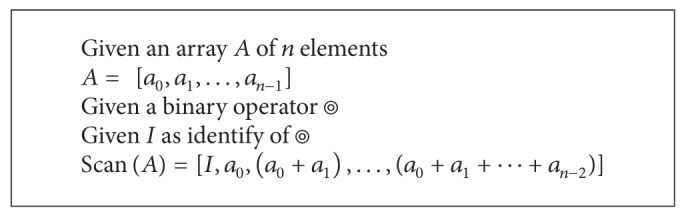
The definition of prefix-sum.

**Algorithm 2:**
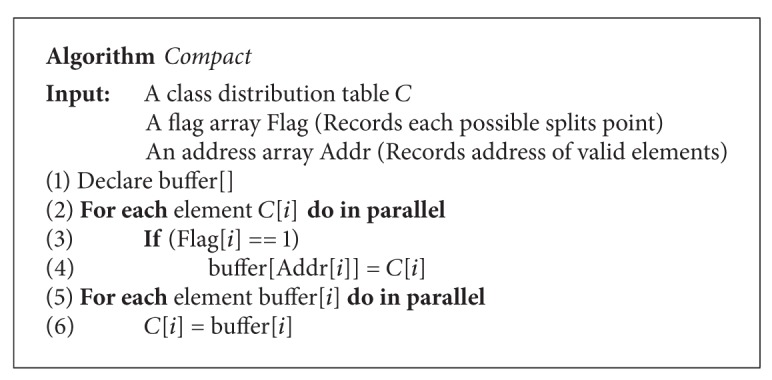
Algorithm of compact function.

**Algorithm 3:**
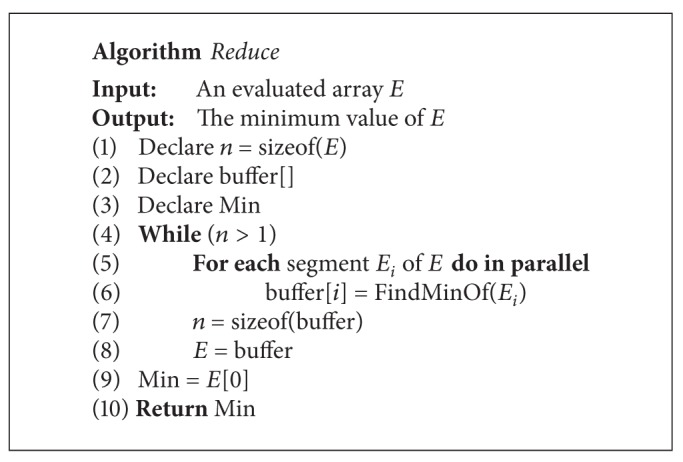
Algorithm of reduce function.

**Algorithm 4:**
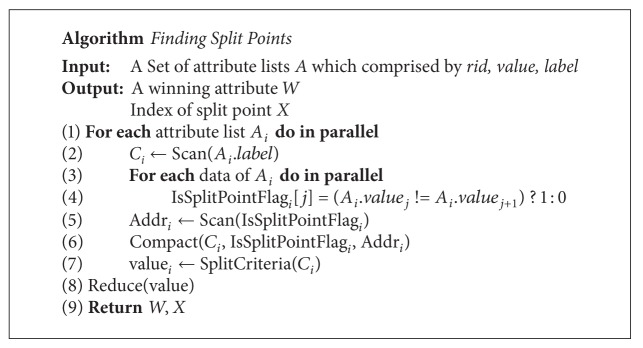
Algorithm of finding split point function.

**Algorithm 5:**
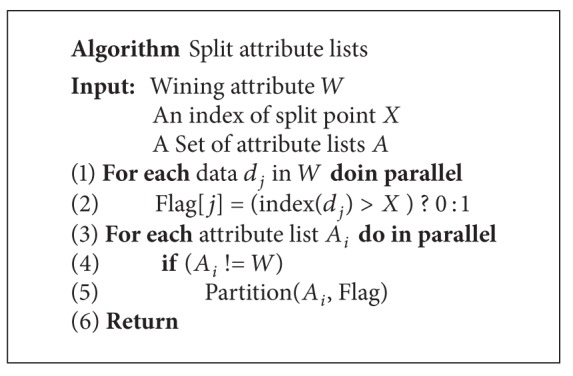
Algorithm of split attribute lists.

**Algorithm 6:**
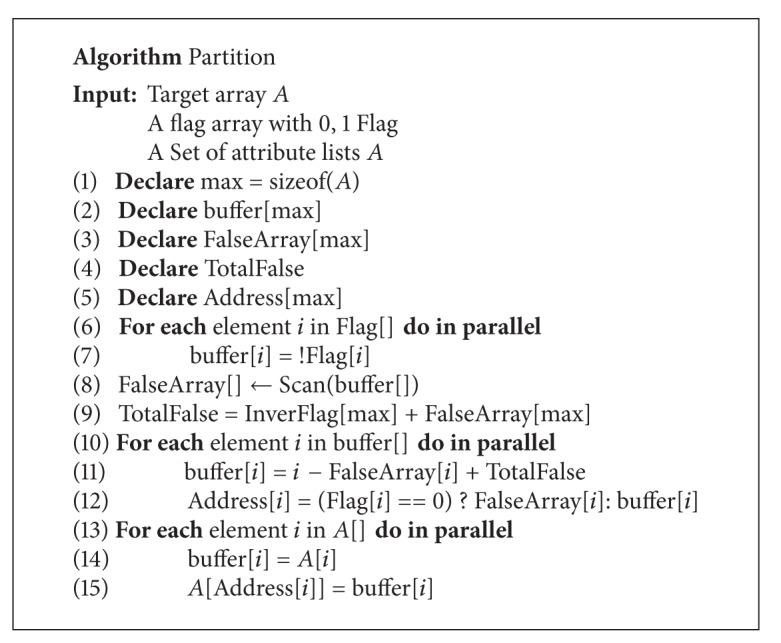
Algorithm of partition.

**Table 1:** Test data set.

	Spambase	Magic Gamma Telescope	MiniBooNE particle identification
Number of attributes	58	11	51
Number of data	4601	19020	130065
Attribute type	Continuous	Continuous	Continuous
Number of classes	2	2	2
Source	UCI	UCI	UCI

**Table 2:** Results of spambase.

Spambase	Weka-j48	SPRINT	CUDT
Time	715 ms	1861.55 ms	124.78 ms
Accuracy	98.32%	97.82%	97.82%
Tree size	379	385	385
Leaf node size	190	193	193

**Table 3:** Results of Magic04.

Magic04	Weka-j48	SPRINT	CUDT
Time	135 ms	409.78 ms	257.72 ms
Accuracy	90.6%	93.54%	93.54%
Tree size	707	1579	1579
Leaf node size	354	790	790

**Table 4:** Results of MiniBooNE.

MiniBooNE	Weka-j48	SPRINT	CUDT
Time	141000 ms	47391 ms	2451.85 ms
Accuracy	98.52%	98.31%	98.31%
Tree size	6441	8127	8172
Leaf node size	3221	4064	4064

**Table 5:** Comparison of SPRINT and CUDT.

Data Sets	Spambase		Magic04		MiniBooNE	
Algorithm	SPRINT	CUDT	SPRINT	CUDT	SPRINT	CUDT
(1) Initiate device	—	1761.4	—	1686.25	—	2106.58
(2) Create attribute lists	1719.71	37.27	57.33	9.4	29270.56	192.45
(3) Finding split point	56.75	58.91	275.06	191.40	11022.46	1479.01
(4) Split attribute lists	93.02	29.02	69.89	63.23	2973.29	726.78
(5) Building phase (3 + 4)	141.83	87.51	352.45	248.31	18120.47	2349.40
(6) Total time (2 + 3 + 4)	1861.55	124.78	409.78	257.72	47391.04	2541.86
